# Reducing the risk of perinatal depression using an app-based cognitive behavioral therapy program: protocol of a randomized controlled trial

**DOI:** 10.3389/fpsyt.2025.1544753

**Published:** 2025-04-14

**Authors:** Li Tang, Hua Qing, Hong Li, Chunfeng Liu, Haijin Wang, Yao Sun, Qian Tan, Yanqiong Wu, Yang Xiao, Jianying Lai, Ling Wang, Li Zhong, Fei Huang, Chunrong Li

**Affiliations:** ^1^ Chengdu Women’s and Children’s Central Hospital, School of Medicine, University of Electronic Science and Technology of China, Chengdu, China; ^2^ Department of Obstetrics and Gynecology, Jintang County Maternity and Child Health Hospital, Chengdu, China; ^3^ Department of Product Engineering, Shanghai Thoven Intelligent Technology Co., Ltd., Shanghai, China; ^4^ Department of Medicine, Chengdu New Genegle Biotechnology Co., Ltd., Chengdu, China

**Keywords:** cognitive behavioral therapy, perinatal depression, prevention, randomized controlled trial, digital technology

## Abstract

**Introduction:**

Cognitive behavioral therapy (CBT) is recognized as one of the most effective methods for reducing the risk of perinatal depression. However, the traditional face-to-face delivery format limits its accessibility. With the advent of digital technology, app-based CBT offers new possibilities, yet its preventive role in perinatal depression remains insufficiently explored. This study aims to determine if pregnant women using an app-based CBT program report reduced depressive and anxious symptoms compared to a control group.

**Methods:**

A two-arm parallel randomized controlled trial of 290 pregnant women will be conducted at Jintang County Maternity and Child Health Hospital in Chengdu, China. Eligible participants who consent to participate will be recruited at 20-24 weeks of gestation and randomly assigned to either the intervention group, which will complete an 8-week mobile app-based CBT program (CareMom), or a control group, which will receive an attention-matched 8-week relaxation training program, also delivered via a mobile app. Randomization will be performed using a computer-generated random number sequence. Primary outcomes include changes in scores on the Edinburgh Postnatal Depression Scale, the Patient Health Questionnaire-9 and the Generalized Anxiety Disorder 7-item from baseline to 6 weeks postpartum. Outcome analyses will be performed on both the intention-to-treat and per-protocol populations.

**Discussion:**

This trial evaluates an app-based CBT program for reducing the risk of perinatal depression. Improved maternal mental health not only benefits the mothers themselves but also may facilitate more optimal parenting behaviors, which could exert a positive influence on the cognitive, emotional, and behavioral development of the infant.

**Clinical Trial Registration:**

https://clinicaltrials.gov/study/NCT06672432, identifier NCT06672432.

## 1 Introduction

The perinatal period represents a critical transition into motherhood, marked by inevitable social, psychological, and hormonal changes that increase susceptibility to mental health disorders such as perinatal depression (1). According to the Diagnostic and Statistical Manual of Mental Disorders Fifth Edition (2), perinatal depression includes major depressive episodes occurring during pregnancy (antenatal depression) or within four weeks postpartum (postpartum depression). However, given that many depressive episodes tend to emerge beyond four weeks after delivery, postpartum depression is typically defined as the presence of depressive symptoms occurring up to 12 months after childbirth in the clinical and research settings (3).

Perinatal depression is common worldwide, with recent reviews estimating its prevalence rate to be between 12% and 25% (4, 5). In China, 16.3% of women were estimated to have symptoms or a diagnosis of perinatal depression (6). However, the treatment rate for major depressive episodes, particularly in psychiatric hospitals, was low at under 20% across the country (7). This low treatment rate may be due to several factors, including a lack of available mental health services, low socioeconomic status, and psychiatric stigma and discrimination (7). Perinatal depression, if left untreated, can lead to serious health consequences for both mother and offspring. Antenatal depression has been linked to delayed fetal development, preterm birth, low birthweight, poor maternal sleep, and an increased risk of postpartum depression (8). Mothers with postpartum depression often interact and communicate less with their infants, have lower rates of breastfeeding initiation and shorter breastfeeding duration, and their children may face delays in emotional, cognitive, and behavioral development (9, 10).

According to a recommendation statement of US Preventive Services Task Force (USPSTF), counseling interventions, such as cognitive behavioral therapy (CBT) and interpersonal therapy, are effective in preventing perinatal depression (11). CBT emphasizes that positive transformations in mood and behavior can be attained through the identification and management of negative thoughts, beliefs, and attitudes, as well as by enhancing positive experiences and activities. CBT provides people with strategies and tools to identify and modify maladaptive cognitive patterns, and behavioral activation.

CBT traditionally delivered in face-to-face formats, either individually or in group settings. With rapid advancement of internet and information technology, researchers have begun to assess the effectiveness of digital versions of CBT during the perinatal period. However, most studies have focused on its therapeutic applications rather than its preventive potential for antenatal and/or postpartum depression (12), leaving the preventive effects largely inconclusive. One large prevention study found that an internet-delivered CBT (iCBT) program, implemented during the second trimester, was ineffective in preventing new onset of perinatal depressive episodes. The authors suggested that iCBT might only be effective in preventing perinatal depression among pregnant women with subthreshold depressive symptoms (13). However, the preliminary evaluations of an app-based CBT program have shown promising reductions in depressive symptoms among postpartum Chinese mothers in the early postpartum period (14).

Given the importance of preventing perinatal depression starting in the antenatal period, we propose that implementing our CareMom program during the second trimester may effectively reduce subsequent depressive symptoms. Therefore, this randomized controlled trial (RCT) aims to evaluate the effectiveness of CareMom, an app-based CBT program, in reducing the risk of perinatal depression among Chinese mothers. We hypothesize that participants using CareMom will experience (1): lower depressive and anxious symptoms at the end of intervention, and (2) lower depressive and anxious symptoms at six weeks postpartum, compared to those receiving a relaxation course. The main objective of our study is to investigate postnatal depression, with the primary outcomes being the changes in depressive and anxious symptoms at six weeks postpartum.

## 2 Methods and analysis

### 2.1 Study design and setting

**Figure 1:**
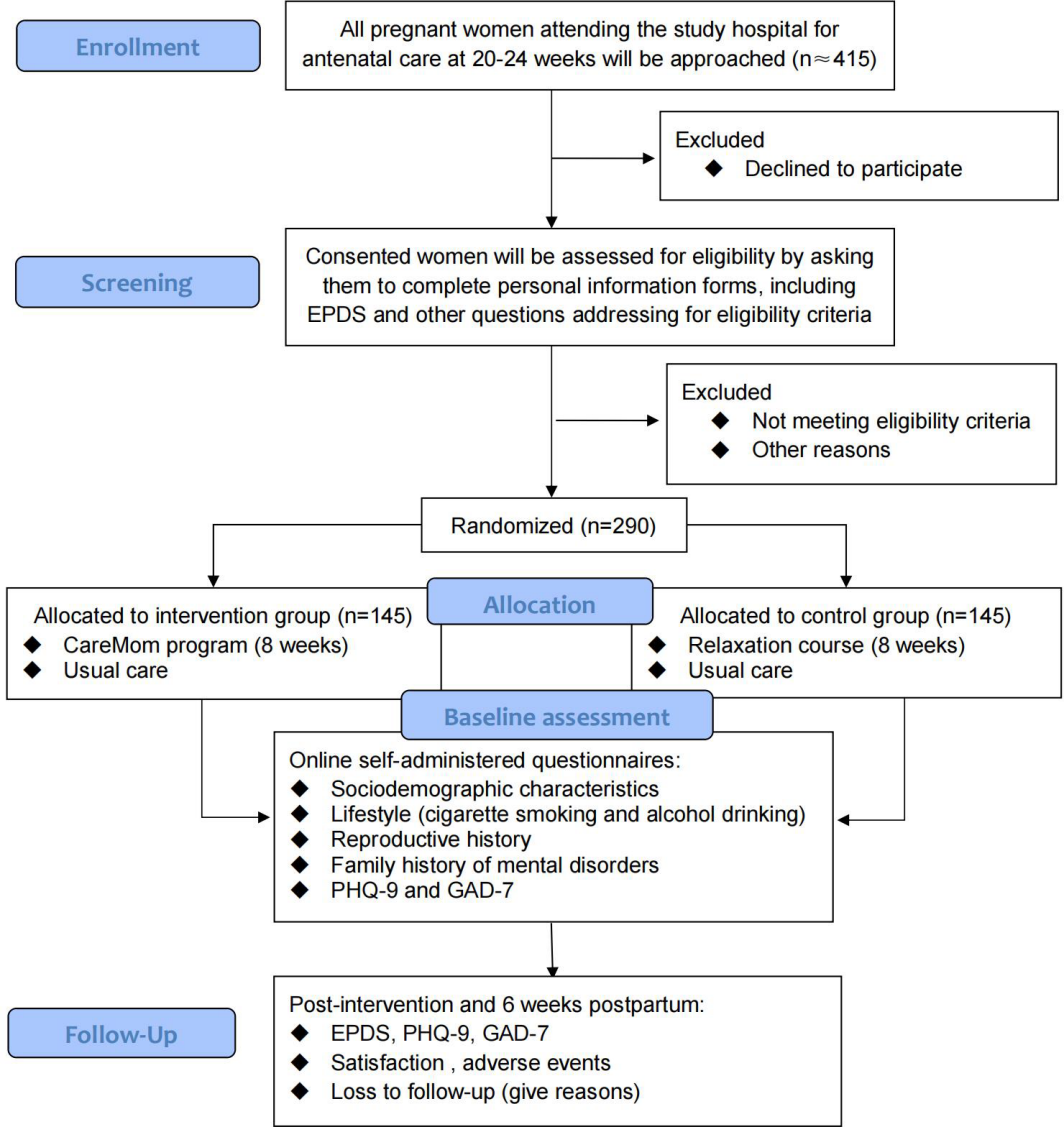
Flow diagram of the RCT.

A two-arm, parallel RCT of 290 participants (145 in each arm) will be conducted at a public maternity hospital in Chengdu. Chengdu, the capital of Sichuan Province in southwest China, has a population of 15.9 million and a disposable annual income per capita of 47,948 RMB in 2022, exceeding the national average of 36,883 RMB. Eligible pregnant women who consent to participate will be recruited from the antenatal clinic of the maternity hospital between 20 and 24 weeks of gestation and will be randomly assigned to either the intervention or active control group using a computer-generated random number. Participants recruitment started in November 2024 and is expected to end in February 2025. We followed the Standard Protocol Items: Recommendations for Interventional Trials (SPIRIT) guidelines for developing our trial protocol (15, 16). The design of the RCT is summarized in **Figure 1**.

### 2.2 Participants

Pregnant women meeting the following inclusion criteria will be invited to participate in the study: (1) aged 18-45 years, (2) at 20-24 weeks of gestation, (3) at risk of depression but not experiencing severe depressive symptoms at recruitment, as indicated by an Edinburgh Postnatal Depression Scale (EPDS) score ranging from 5 and 12 (17), (4) own a smartphone, and (5) are able to independently engage with the program.

Pregnant women will be excluded from the study if they meet any of the following criteria: (1) have serious medical conditions or pregnancy complications that may affect their psychological condition, as determined by their medical doctor, (2) have a prior diagnosis of any mental disorders, (3) are currently or were recently (within the past six months) receiving any kinds of psychological services or treatments, (4) have a history of self-harm or suicide, (5) have any suicidal thoughts in the past 12 months, (6) experienced fetal deaths in the past 18 months.

### 2.3 Recruitment and randomization

The participants will be recruited at the antenatal clinic. The researchers, who are obstetricians at the research hospital, will introduce the aims (i.e. to improve mothers’ mental health) and procedures of the study to potentially eligible women both verbally and through an information sheet. If a woman consents to participate, she will complete the consent and personal information forms. The personal information forms include the EPDS, along with questions addressing other eligibility criteria for the study, such as age, gestational week, pregnancy complications, history of mental disorders, etc. After reviewing the personal information, the researchers will then allocate eligible participants to either the intervention group (the CareMom group) or the active control group (the relaxation training group) using a computer-generated random number. The random numbers range from 0 to 1. If the number is greater than 0.5, the participant will be allocated to the CareMom group; otherwise, she will be allocated to the active control group.

### 2.4 Data collection and measurements

We have developed online self-administered questionnaires using the survey platform WJX.CN (https://www.wjx.cn/), with corresponding QR codes generated for easy access. The schedule of measurements is present in **Table 1**.

**Table 1:** Measurements to be taken at each point in trial.

	Study period			
	Enrolment	Allocation	Post-allocation	
		T_0_ (Day 0)	T_1_ (8 weeks)	T_2_ (6 weeks postpartum)
Informed consent	**×**			
Basic personal information (e.g. age, pregnancy complications, history of mental disorders)	**×**			
EPDS	**×**		**×**	**×**
Sociodemographic characteristics		**×**		
Reproductive history		**×**		
Lifestyle (cigarette smoking and alcohol drinking)		**×**		
Family history of mental disorders		**×**		
PHQ-9		**×**	**×**	**×**
GAD-7		**×**	**×**	**×**
CSQ-8			**×**	

EPDS, Edinburgh Postnatal Depression Scale; PHQ-9, Patient Health Questionnaire-9; GAD-7, Generalized Anxiety Disorder 7-item; CSQ-8, Client Satisfaction Questionnaire; **×**, measurements to be taken at this point.

Following randomization, all participants will be asked to scan a QR code to complete a structured baseline questionnaire, collecting data on sociodemographic characteristics (i.e. marital status, occupation, educational level, and family income), reproductive history, lifestyle factors (i.e. cigarette smoking and alcohol drinking), and family history of mental disorders. In addition to the EPDS which is collected at recruitment (18), we will also use the Patient Health Questionnaire-9 (PHQ-9) (19) and the Generalized Anxiety Disorder 7-item (GAD-7) (20) to evaluate participants’ depressive and anxiety symptoms at baseline. Trained nurses will be available for classification if needed.

The EPDS has been widely employed in antenatal and postnatal depression research to measure depressive symptoms over the past seven days (17). It consists of 10 items rated on a four-point scale from 0 to 3, with a maximum score of 30, where higher scores indicate more severe depressive symptoms. We will use the Chinese version of EPDS in this study (21). For Chinese women, EPDS scores above 9 suggest a high likelihood of depression (21, 22) and scores above 15 indicate severe depression (23).

The PHQ-9 is a widely used self-administered instrument for diagnosing and assessing the severity of depression. Patients rate the frequency of specific depressive symptoms experienced over the past two weeks on a four-point scale, ranging from “not at all” to “most days”. Total scores range from 0 to 27, with a score of 10 or greater being the most common cut-off for screening major depressive disorder (19). Studies conducted in mainland China have validated the reliability and validity of the Chinese version of the PHQ-9 in detecting major depression (24, 25).

The GAD-7 is a valid self-assessment tool used to measure anxiety symptoms over the past 14 days. It consists of seven items, each rated on a score from 0 to 3. The total score ranges from 0 to 21, with higher scores reflecting more severe anxiety symptoms. Scores between 5 and 9 suggest mild anxiety, and a score greater than 14 indicates severe anxiety (20). In our study, the Chinese version of GAD-7 is applied (26).

The depressive and anxiety symptoms will be measured at follow ups, with all participants asked to complete the EPDS, PHQ-9 and GAD-7 after the 8-week intervention period, and again at 6 weeks postpartum. The use of both EPDS and PHQ-9 allows for a more comprehensive assessment of depressive symptoms, as EPDS is specifically designed for perinatal populations and captures unique features such as anxiety and guilt, while PHQ-9 aligns with DSM-based diagnostic criteria, facilitating comparisons with broader clinical and research settings. This dual approach ensures a more nuanced evaluation of depression severity and symptom patterns in our study population. In addition, an acceptability survey will be administered to assess participant satisfaction level post-intervention. For participants who do not complete the project, their reasons for withdrawal will be gathered through a brief questionnaire.

In this study, we will use the Client Satisfaction Questionnaire (CSQ-8) to assess participant satisfaction with the intervention (27). The CSQ-8 is a widely used, standardized tool designed to measure client satisfaction with healthcare services and interventions. It consists of 8 items, each scored on a Likert scale, with higher scores indicating greater satisfaction. The questionnaire covers aspects such as perceived quality of service, the extent to which the service met participants’ needs, and their overall satisfaction with the experience.

All unfavorable or unintended events affecting participants in the study, such as physical discomfort, psychological distress, or any other negative experiences related to the intervention, will be recorded using a pre-specified adverse event questionnaire. Participants are encouraged to report any discomfort they experience, and all reported events will be documented and monitored throughout the study period.

Any serious adverse events will be promptly reported to the safety board of the trial, who will then evaluate the risks and provide recommendations to the research team on whether to continue, temporarily pause, or discontinue the trial due to any increased risk or significant adverse events. The safety board includes three experts specializing in clinical trial conduct, statistics, and mental health. The trial manager will compile a report detailing the number of adverse event notifications at each assessment period for review by the safety board.

### 2.5 Interventions

Immediately after baseline data collection, all participants will be asked to scan a Quick Response (QR) code to open a mini program in the WeChat application.

The intervention program, CareMom, is a structured, 8-week digital intervention delivered through a WeChat mini-program in Chinese, developed by Shanghai Thoven Technology Co. Ltd., China, under the guidance of psychologists and psychiatrists. CareMom is designed to support perinatal mental health by providing daily challenges, educational content, and interactive exercises that target cognitive restructuring, behavioral activation, and worry management, key therapeutic components for alleviating symptoms of depression and anxiety.

**Figure 2:**
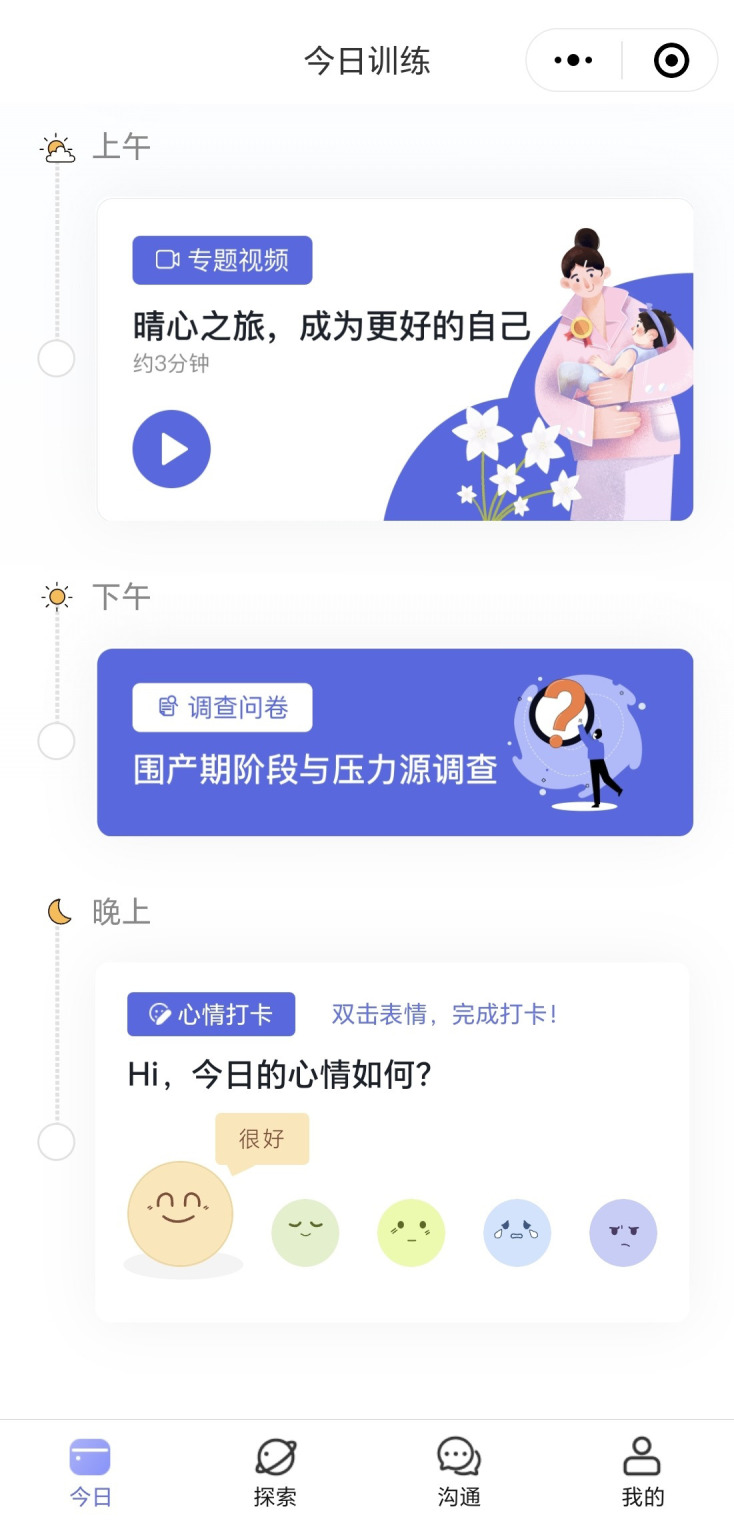
Screenshot of a daily task list within CareMom.

The intervention is divided into daily tasks spread across eight weeks, with each day involving a variety of interactive elements such as videos, text-based content, guided reflections, and mental health check-ins. Upon logging into the system, participants are directed to a personalized dashboard page displaying their daily tasked need to complete (see **Figure 2**). If the user misses a daily challenge, she can complete that challenge in the later days. Each week has a distinct focus that progressively builds the user’s skills and understanding in managing emotional health. Tasks are designed to be short yet impactful, aiming to enhance engagement and adherence. **Table 2** describes the weekly focus of the 8-week intervention.

**Table 2:** Description of the 8-week intervention program.

Week	Focus	Description
Week 1	Emotional Awareness	Participants engage in mood tracking, emotion identification, and gratitude journaling.
Week 2	Cognitive Patterns and Automatic Thoughts	Introduces participants to cognitive distortions and automatic thoughts.
Week 3	Core Beliefs and Cognitive Restructuring	Guides participants to identify negative core beliefs, challenge them, and replace them with positive ones.
Week 4	Anxiety Management Techniques	Focuses on anxiety management through techniques like progressive muscle relaxation, controlled breathing exercises, and understanding the physiological responses associated with anxiety.
Week 5	Advanced Cognitive Techniques for Worry	Builds on cognitive restructuring by addressing specific cognitive distortions like catastrophic thinking.
Week 6	Exposure to Feared Thoughts and Situations	Uses guided imagery and exposure-based exercises to help users confront distressing thoughts or worries gradually, with the aim of reducing avoidance behavior.
Week 7	Behavioral Activation for Positive Reinforcement	Introduces behavioral activation techniques, encouraging users to engage in enjoyable and purposeful activities.
Week 8	Consolidation and Long-Term Management	Users review and consolidate skills learned throughout the program, focusing on relapse prevention strategies and setting goals for maintaining emotional well-being beyond the intervention.

**Figure 3:**
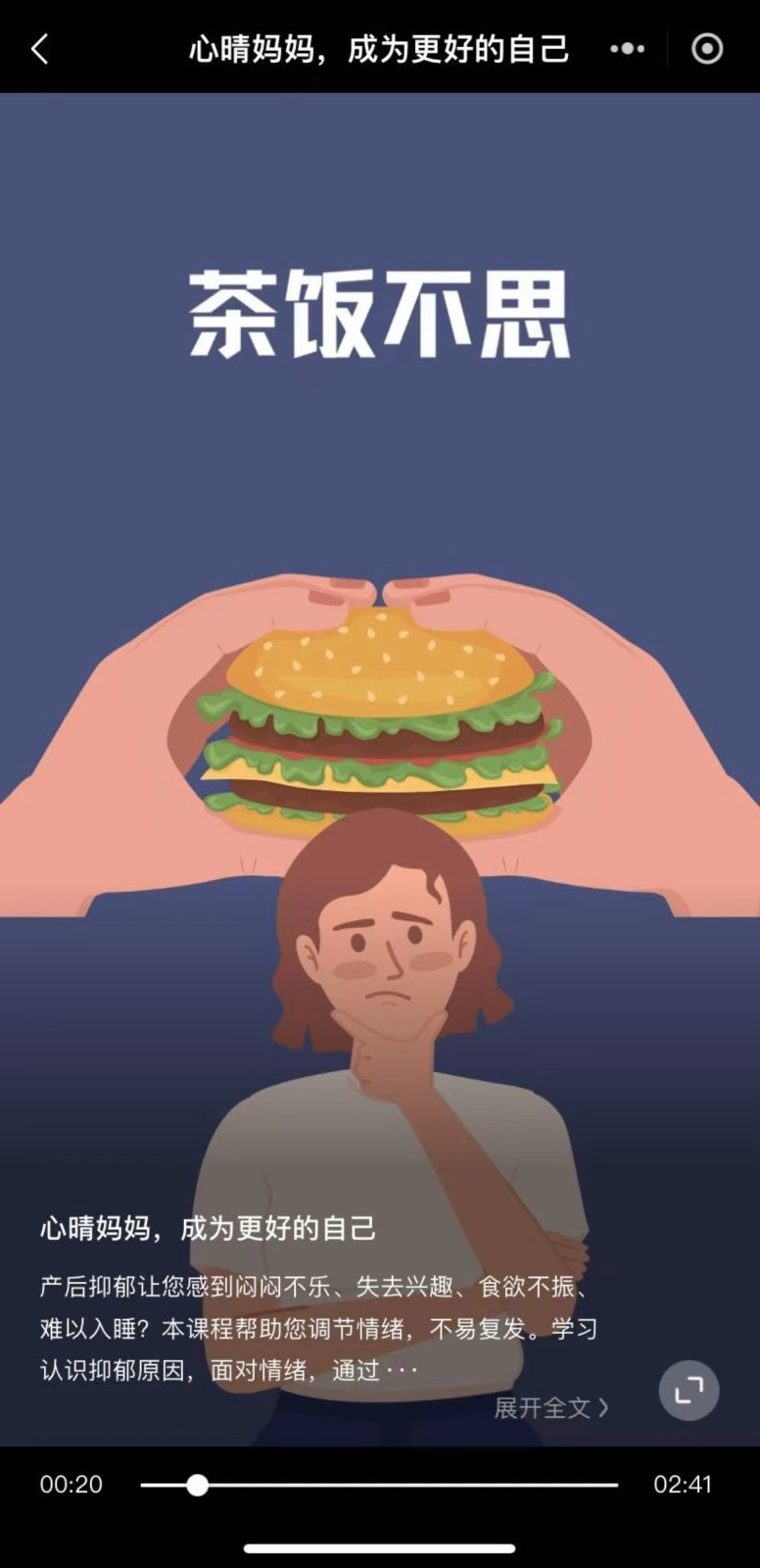
Screenshot of a video for psychological education.

**Figure 4:**
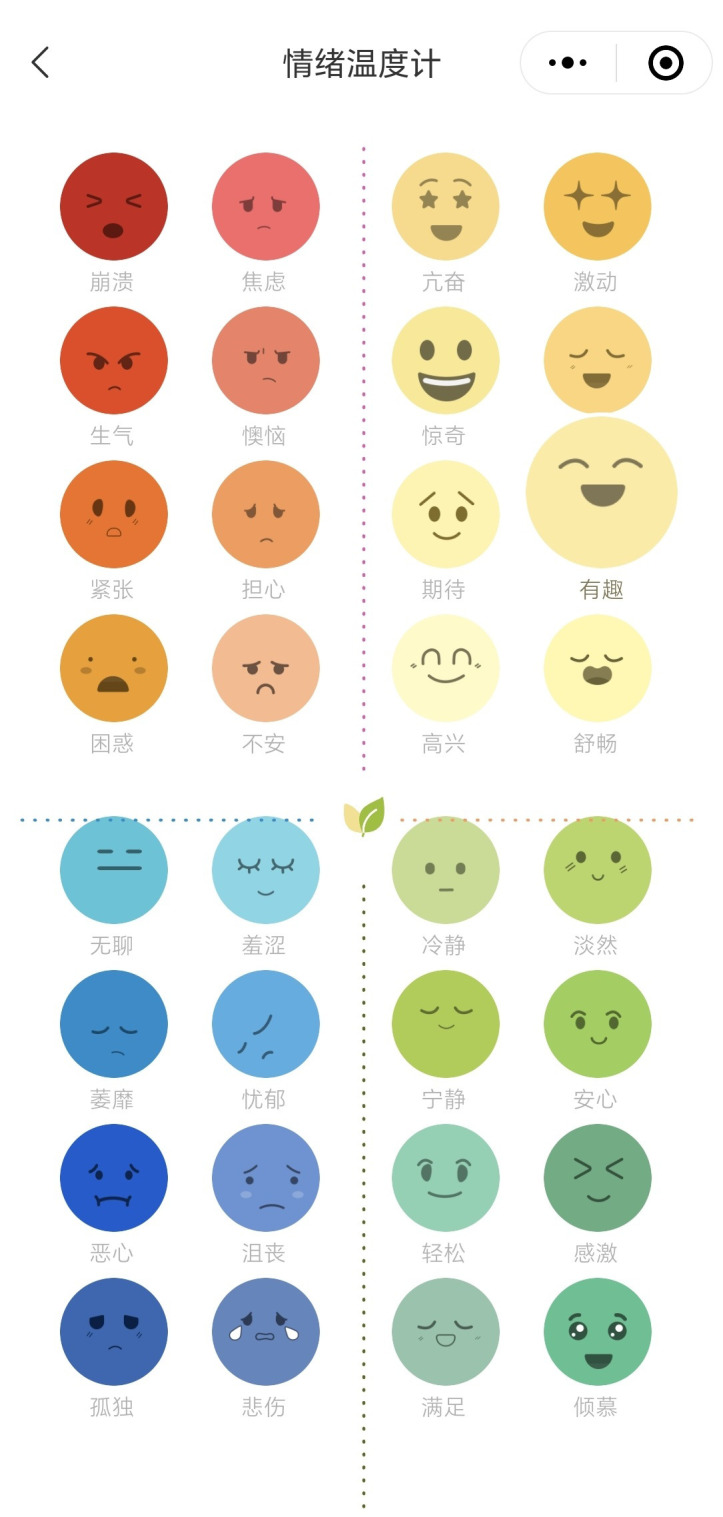
Screenshot of the mood checker tool.

**Figure 5:**
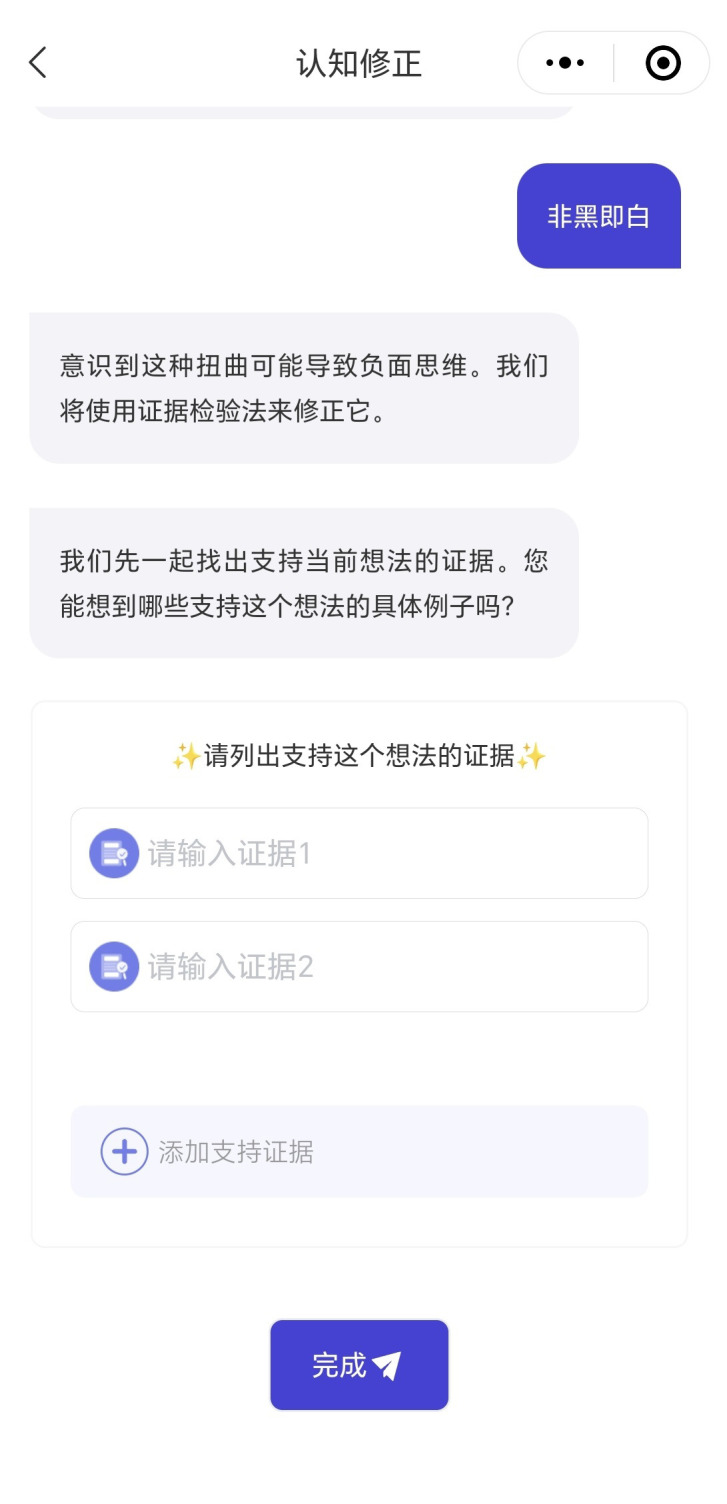
Screenshot of the though challenger tool.

**Figure 6:**
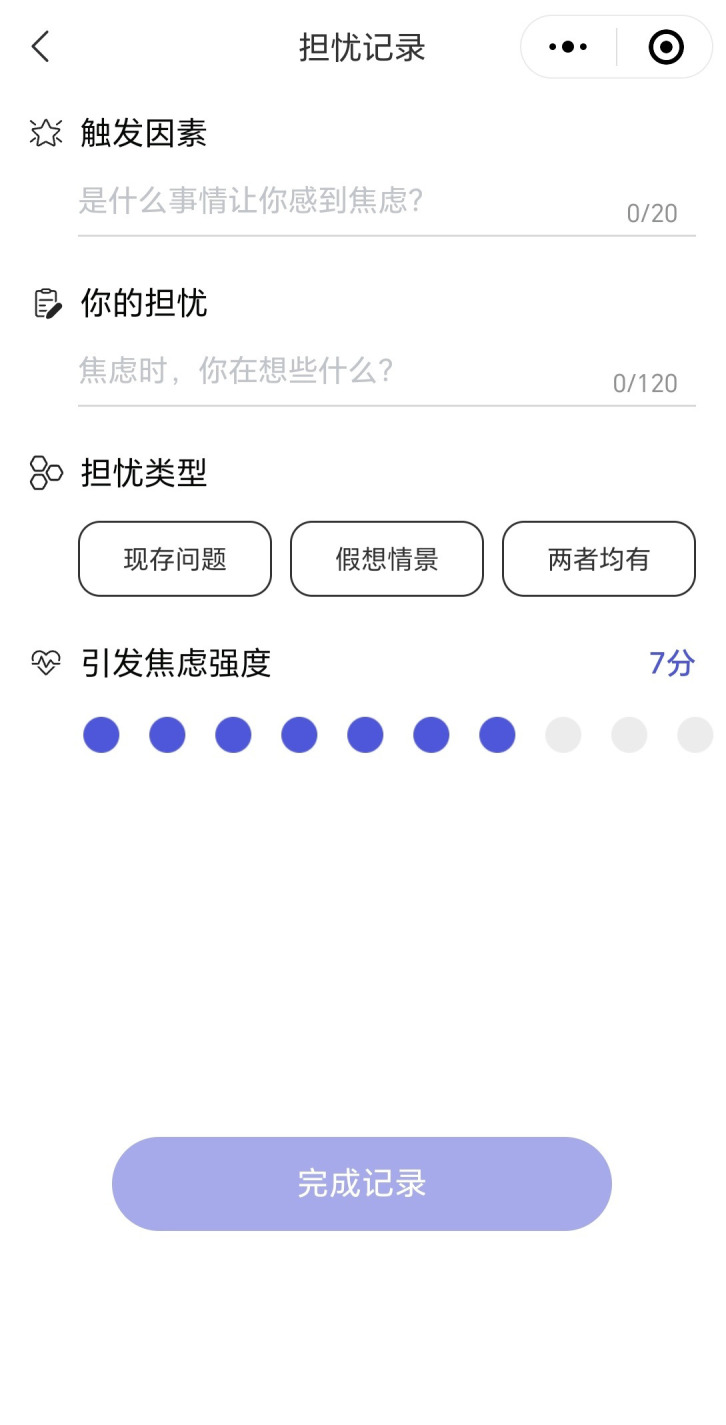
Screenshot of the worry recording tool.

CareMom presents users with their daily tasks, which they are required to complete each day. These tasks typically include key components such as video-based psychoeducation (see **Figure 3**) and a mood checker (see **Figure 4**). Some tasks will automatically trigger interactive tools designed to help users complete specific tasks more effectively. For example, in Week 2, when users engage in cognitive restructuring, the Thought Challenger tool is triggered to assist them in identifying and challenging Automatic Negative Thoughts (see **Figure 5**). Similarly, during Week 3 on Anxiety Management Techniques, a worry-recording tool is activated to guide users in documenting and analyzing their worries (**Figure 6**).

Throughout the eight-week intervention, CareMom offers a range of additional tools tailored to different therapeutic goals. These include an activity list builder to support behavioral activation, a postpone worries tool for managing anxious thoughts, and tools for imaginal exposure and behavioral experiments to facilitate gradual desensitization and cognitive restructuring. By seamlessly integrating these tools into the daily tasks, CareMom ensures that users receive structured, hands-on guidance in applying intervention techniques to their daily lives.

In attention to the 8-week intervention program, participants in the intervention group will receive standard perinatal care throughout the study. This includes routine prenatal check-ups, postpartum nursing care, and scheduled postnatal visits, ensuring that all participants receive the essential healthcare services typically provided during the perinatal period.

### 2.6 Matched attention control

The control group in this study will participate in an attention-matched, 8-week online relaxation training program delivered through a mobile application. This program is designed to mirror the structure and engagement level of the intervention group, providing participants with a similar amount of interaction and daily activities. Each day, participants in the control group will complete 1-2 daily tasks focused on relaxation techniques, such as breathing exercises, mindfulness meditation, and guided imagery. Each task is brief, approximately 3 minutes in duration, making it easy for participants to incorporate into their daily routine.

**Figure 7:**
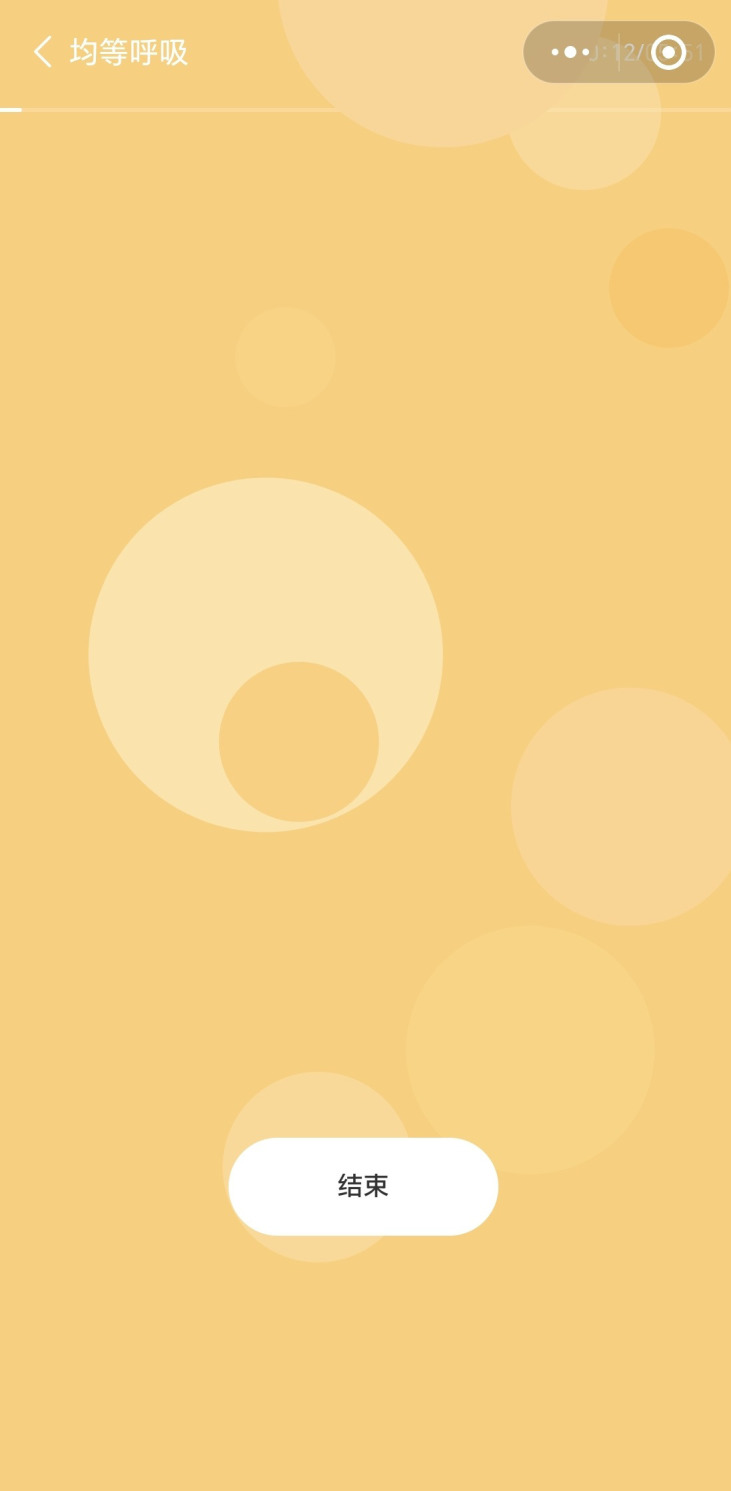
Screenshot of the guided breathing tool.

Similar to the intervention group, participants in the control group will receive a daily task list, where each day’s page presents relaxation-focused tasks. These tasks include breathing exercises, mindfulness meditation, and guided imagery, designed to promote relaxation and stress reduction. Each task is brief, approximately 3 minutes in duration, making it easy for participants to incorporate into their daily routine. CareMom also provides interactive tools tailored to each task. These tools assist users in completing their relaxation exercises more effectively. For example, CareMom includes a tool that guides users through their breathing exercises, ensuring they follow the technique correctly for maximum benefit (see **Figure 7**). Similar tools are integrated throughout the program to support various relaxation techniques, helping participants engage more deeply with the intervention.

User engagement and adherence in both the intervention and control groups are automatically tracked by the system, which records key metrics such as daily task completion, login frequency, module progression, and time spent on each activity. To minimize any potential adverse effects of breathing exercises (28), a cautionary statement was included in an appropriate location within the app: “Should you experience any discomfort during the breathing exercises, please stop immediately. If the discomfort persists, we recommend consulting your obstetrician.”

### 2.7 Outcomes

Primary outcomes include changes in EPDS, PHQ-9 and GAD-7 scores from baseline (T_0_) to 6 weeks postpartum (T_2_).

The secondary outcomes include: (i) changes in EPDS, PHQ-9 and GAD-7 scores from baseline (T_0_) to post-intervention (T_1_), and (ii) comparisons of CSQ-8 results and the number of adverse events post-intervention (T_1_) between the intervention and control groups.

### 2.8 Blinding

Blinding procedures are implemented for both participants and trial staff to minimize potential bias and ensure the validity of the study results.

For participants, blinding is achieved by providing all individuals with the same QR code to access the mini program. Upon scanning, participants are randomly assigned to either the intervention or control content within the same program interface, without any indication of their group allocation. Both intervention and control participants access the same mini program, with only the internal content differing based on group assignment. This setup ensures that participants remain unaware of their specific allocation, thus preserving blinding at the participant level.

For trial recruitment and management staff, including obstetricians, nurses, and the statistician responsible for data analysis, blinding is also maintained to prevent any inadvertent influence on study outcomes. Trial staff are blinded to each participant’s group assignment. All study results will be analyzed and interpreted in a blinded manner to reduce the risk of biased interpretation.

### 2.9 Sample size determination

In this study, to detect a medium effect size (d=0.37) for a two-tailed independent t-test at a significance level of 5% with a power of 80%, at least 115 participants are required for each group (29). Taking into account a loss to follow-up of 20% in our study, the total sample size required at baseline for the RCT is 290 (145 per group). It is assumed that approximately 30% of women will decline or be ineligible to participate; we plan to approach at least 415 pregnant women at the hospital antenatal clinic. G*Power software was used for the sample size calculation.

### 2.10 Statistical analysis

We will descriptively summarize participants’ baseline characteristics, outcome variables, satisfaction levels and adverse events. Independent t-tests or Mann-Whitney U tests will be performed to compare continuous variables, and chi-square or Fisher’s exact tests will be applied for categorical characteristics. To account for the correlation of repeated measures from the same participant, generalized linear models with random effects (participants) will be fitted to evaluate the intervention effect on outcome variables over time, adjusting for potential confounders such as age, parity, and family history of mental disorders.

Outcome analyses will be conducted on both the intention-to-treat population, which includes all randomized participants, and the per-protocol population, consisting of eligible participants who adhere to the planned intervention and follow ups. Missing data for primary and secondary outcomes will be imputed using the last value carried forward method in the intention-to-treat analysis. Additionally, to assess the impact of intervention adherence, adherence metrics—such as daily task completion rates and module progression—will be incorporated into sensitivity analyses. Participants will be stratified based on their engagement levels, and adherence data will be included as exploratory covariates in the regression models. These analyses will help determine whether engagement level influences intervention effectiveness and whether adherence plays a moderating role in treatment outcomes.

### 2.11 Monitoring

We have developed a data monitoring committee (DMC) to monitor conduct and quality of the trial. The DMC includes four independent members with expertise in maternal health, psychology, biostatistics and trial methodology. The DMC meeting will take place every three months.

## 3 Discussion

The present trial aims to address the existing research gap by assessing the preventive effects of the CareMom program, an app-based CBT intervention tailored specifically for perinatal mental health. By delivering CBT through a mobile platform, CareMom can reach pregnant women who may otherwise lack access to mental health resources. This could potentially increase the utilization of preventive mental health services during the perinatal period, particularly in areas with limited healthcare access or among women facing social or time-related barriers. If shown to be effective, CareMom could serve as a scalable solution to bridge existing mental health service gaps and offer an accessible form of mental health support.

To ensure methodological rigor, we have carefully designed this RCT and include comprehensive details about our implementation plan to provide reliable evidence for the effects of the CareMom program. Specifically, we will include a control group receiving a placebo intervention, acknowledging the importance of the placebo effect in psychological interventions. This approach will help differentiate genuine preventive benefits from those shaped by participants’ expectations. In addition, we have incorporated blinding procedures for both participants and staff involved in the trial. This minimizes the risk of performance and detection biases, enhancing the validity and generalizability of our findings.

Our study has some limitations. Firstly, this study will be conducted in a district hospital located in a large urban area, and the sociodemographic characteristics of the participants may differ from those of mothers in smaller cities or rural regions. Secondly, although the EPDS cut-off scores used in the selection criteria are based on existing evidence, the potential for false negatives remains, which may affect the representativeness of our sample. In addition, participants will be followed up only until six weeks postpartum, whereas postpartum depression can occur within 12 months following delivery (3). Therefore, further investigations covering a wider range of postpartum periods would be beneficial. Given that this is our first study testing the effectiveness of an i-CBT program for reducing the risk of perinatal depression, we adopted a structured, generalized intervention approach. However, future refinements will incorporate user feedback on factors such as compliance, convenience, and effectiveness to enhance the program, ultimately allowing for a more individualized approach that better targets specific concerns and improves overall efficacy. Lastly, the intervention relies on digital engagement, which may not suit all user preferences; technological challenges, such as internet access or device familiarity, could impact adherence and outcomes, particularly among populations less accustomed to using mobile applications.

In summary, we will conduct a definitive RCT to evaluate whether the CareMom, an app-based CBT program, yields better outcomes than a relaxation course in reducing the risk of perinatal depression among 290 Chinese mothers. We will employ rigorous methodologies to minimize bias and establish several working committees to ensure the quality of the trial. The findings from this study could have important implications for the prevention of perinatal depression in China.

## 4 Ethics and dissemination

Ethics approval has been granted by the Ethics Committee of Jintang County Maternity and Child Health Hospital (Approval number: 20240511-1). Signed informed consent will be obtained from all participants, and confidentiality of collected information will be strictly maintained throughout the study. The trial database will be de-identified, password protected and accessible only to the research team. Identifiable data will be available exclusively to authorized study investigators on a need-to-know basis for study-related purposes, such as ensuring appropriate follow-up. Aggregated data and study findings will be presented at national and international academic conferences and disseminated in peer-reviewed journals.
